# Uncertainty and the Value of Information in Risk Prediction Modeling

**DOI:** 10.1177/0272989X221078789

**Published:** 2022-02-25

**Authors:** Mohsen Sadatsafavi, Tae Yoon Lee, Paul Gustafson

**Affiliations:** Respiratory Evaluation Sciences Program, Collaboration for Outcomes Research and Evaluation, Faculty of Pharmaceutical Sciences, The University of British Columbia, Vancouver, Canada; Respiratory Evaluation Sciences Program, Collaboration for Outcomes Research and Evaluation, Faculty of Pharmaceutical Sciences, The University of British Columbia, Vancouver, Canada; Department of Statistics, The University of British Columbia, Vancouver, Canada

**Keywords:** Bayesian statistics, decision theory, predictive analytics, precision medicine, value of information

## Abstract

**Background:**

Because of the finite size of the development sample, predicted probabilities from a risk prediction model are inevitably uncertain. We apply value-of-information methodology to evaluate the decision-theoretic implications of prediction uncertainty.

**Methods:**

Adopting a Bayesian perspective, we extend the definition of the expected value of perfect information (EVPI) from decision analysis to net benefit calculations in risk prediction. In the context of model development, EVPI is the expected gain in net benefit by using the correct predictions as opposed to predictions from a proposed model. We suggest bootstrap methods for sampling from the posterior distribution of predictions for EVPI calculation using Monte Carlo simulations. We used subsets of data of various sizes from a clinical trial for predicting mortality after myocardial infarction to show how EVPI changes with sample size.

**Results:**

With a sample size of 1000 and at the prespecified threshold of 2% on predicted risks, the gains in net benefit using the proposed and the correct models were 0.0006 and 0.0011, respectively, resulting in an EVPI of 0.0005 and a relative EVPI of 87%. EVPI was zero only at unrealistically high thresholds (>85%). As expected, EVPI declined with larger samples. We summarize an algorithm for incorporating EVPI calculations into the commonly used bootstrap method for optimism correction.

**Conclusion:**

The development EVPI can be used to decide whether a model can advance to validation, whether it should be abandoned, or whether a larger development sample is needed. Value-of-information methods can be applied to explore decision-theoretic consequences of uncertainty in risk prediction and can complement inferential methods in predictive analytics. R code for implementing this method is provided.

## Highlights

- Uncertainty in the outputs of clinical prediction models has largely been approached from a purely statistical perspective.
- In decision theory, uncertainty is associated with loss of benefit because it can prevent one from identifying the most beneficial decision.
- This article extends value-of-information methods from decision theory to risk prediction and quantifies the expected loss in net benefit due to uncertainty in predicted risks.
- Value-of-information methods can complement statistical approaches when developing or validating clinical prediction models.

## Introduction

A risk prediction model can be seen as a mathematical function that maps an individual’s characteristics to their predicted risk of an event, enabling risk-stratified treatment decisions. The development of a risk prediction model is typically based on individual-level data from a finite sample. As such, the resulting predictions are inherently uncertain. In practice, uncertainty in predictions is often ignored, and a deterministic function is advertised as the final model. For example, the proposed model can be the set of (penalized) maximum likelihood estimates of coefficients in a classical regression framework or the final state of a machine-learning model such as an artificial neural network. Such determinism in predictions might have stemmed from the need to use the model at the point of care, where it is most practical to make decisions based on a single good estimate of risk. Notwithstanding such practicality, uncertainty in predictions remains relevant: had we used another sample for model development, we could have arrived at a different model, a different predicted value for the patient, and thus potentially a different treatment decision.

The topic of the development sample size in risk prediction is a subject of active research. Recent developments on sample size calculations have focused on meeting prespecified criteria on prediction error^1^ or on overall calibration performance such as mean calibration or the degree of optimism in predictions.^2,3^ The adequacy of the development sample of a given size has also been investigated in terms of the stability of predictions.^4^ Despite targeting different objectives, such approaches are fundamentally concerned with the accuracy of predictions from a purely statistical perspective. Given that risk prediction models are used for patient care, of ultimate relevance is to what extent such uncertainty affects the outcome of treatment decisions. This perspective of prediction uncertainty is not sufficiently investigated.

We are motivated by the approach taken in the field of decision analysis to tackle a similar problem. In informing policy decisions about the adoption of new interventions, decision-analytic (e.g., cost-effectiveness) models are developed that quantify the net benefit (NB) of each competing intervention at the population level.^5^ Such models are based on uncertain input parameters such as treatment effect or costs of disease management. Thus, the resulting NB projections are uncertain. The impact of such uncertainty is that the intervention that is identified as having the highest expected NB might not be the one with the highest true NB. Consequently, uncertainty is associated with an expected loss in NB. The expected value of this loss, termed the expected value of perfect information (EVPI), can be quantified from the output of a probabilistic decision-analytic model.^6^ This approach and its extensions, broadly referred to as value-of-information analysis,^7^ provide a fully decision-theoretic framework for quantifying the impact of uncertainty in health policy making.^8^

In this work, we extend the definition of EVPI from decision analysis to the development phase of risk prediction models, with the aim of quantifying the expected loss in NB due to uncertainty in estimating model parameters from a finite development sample. This provides a decision-theoretic approach to the question that naturally arises after the development of a risk prediction model: whether the model is “good enough” and can advance to the next stage of research, whether it should be abandoned, or whether more evidence is needed to decide.^9,10^

### Net Benefit Calculations for Risk Prediction Models

The NB approach for evaluating the utility of risk prediction models has gained significant popularity because of its rigorous decision-theoretic underpinning as well as its relative ease of calculation.^11^ To turn a continuous predicted risk to a binary action (treat or not treat), one needs to specify a context-dependent treatment threshold on predicted risks. Such a threshold should ideally be informed by the relative weight of clinical consequences of false-positive (harm) versus true-positive (benefit) classifications. Vickers and Elkin showed that this threshold acts as an exchange rate between true- and false-positive outcomes, enabling the calculation of NB.^11^ Imagine a decision maker (e.g., a guideline development team after consulting a patient group about their preferences) concludes that patients with acute myocardial infarction (AMI) should receive a more aggressive treatment if their 30-d risk of mortality is >2% and no such treatments if the predicted risk is <2%. The group is ambivalent between treatment and no treatment if the predicted risk is precisely 2%. Such ambivalence indicates that the decision maker equates the benefits associated with a 2% chance of true positive to be equal to the harms associated with a 98% chance of false positive. This itself means the benefit of a true-positive diagnosis is 49 times the harm of a false-positive diagnosis. This enables the calculation of NB in true-positive units net of harms in false-positive units. Generalizing this approach, at threshold value of z , the NB can be calculated as

$$NB(z)=P(True\;Positive)-P(False\;Positive)\frac{z}{1-z}.$$

Here, z/(1−z) represents the relative weight of a false-positive versus a true-positive classification and thus captures the harm-benefit tradeoff at this threshold. In practice, the NB is often calculated for a plausible range of thresholds.

Imagine we have a proposed model based on a development sample of n independent observations. Let $\pi_{i}\equiv \pi \left(X_{i}\right)$ be the predicted risks for the *i*th patient in this sample with covariate pattern $X_{i}$ , and $Y_{i}$ be the corresponding observed binary outcome. At a threshold value of z , the *i*th patient contributes $I(\pi_{i}>z)Y_{i}$ to the probability of true positive and $I\left(\pi_{i}>z\right)\left(1-Y_{i}\right)$ to the probability of false positive. The NB of the proposed model can be consistently estimated as^11^

$$\hat{{NB}_{Model}}\left(z\right)=\frac{1}{n}\sum_{i=1}^{n}\left\{I\left(\pi_{i}>z\right)\left[Y_{i}-\left(1-Y_{i}\right)\frac{z}{1-z}\right]\right\}.$$

The NB of the model should always be compared with that of at least 2 alternatives: treating none and treating all. We use the “opt-in” definition of NB and set the default decision to be treating no one, with NB = 0.^9^ The decision to treat all is equal to assuming each individual is positive, whose NB can be consistently estimated as

$$\hat{{NB}_{all}}\left(z\right)=\frac{1}{n}\sum_{i=1}^{n}\left\{Y_{i}-\left(1-Y_{i}\right)\frac{z}{1-z}\right\}.$$

If there are preexisting models applicable to this decision context, their NB should also be compared with the NB of the model. However, to facilitate the developments and without loss of generality, we assume the proposed model is the only relevant risk prediction algorithm.

Evaluating a model in the same sample in which it is developed might result in optimistic conclusions about its performance.^12^ A commonly employed method for correcting for such optimism is the Harrell's bootstrap.^13^ This approach involves obtaining a bootstrap sample from the development data set, fitting a new model in this sample, and calculating the NB (or other metrics) for the new model in the same bootstrap sample as well as in the original sample and then recording the difference. Repeating these steps many times and averaging the differences will provide an estimate of optimism. This approach is based on the notion that the difference between the performance of the model in the bootstrap sample and in the original sample is an almost unbiased estimate of the difference between its performance in the original sample and in the generating population.^14^

### A Bayesian Approach toward NB Calculation

Value-of-information analysis is a strictly Bayesian paradigm as it treats the unknown true associations as random entities for which we have partial information.^6^ Here, the random entity of interest is the “correct” (i.e., strongly calibrated^15^) model, indexed by a set of unknown parameters θ, that for the *i*th individual returns the correct risk $p_{\theta i}\equiv p(X_{i},\theta )$ , the average risk among all individuals with the same covariate pattern $X_{i}$ . Let P(θ|D) be the posterior joint probability density function of model parameters that represents our knowledge about the parameter values of the correct model after observing the development data D . The Bayes’s rule P(θ|D)∝P(θ)P(D|θ) indicates that our knowledge is influenced by the information from the development sample ( P(D|θ) ) and any prior knowledge on the correct model ( P(θ) ).

The crucial next step is to recognize that if the correct risks are available, we can replace the observed response $Y_{i}$ with the correct risk $p_{\theta i}$ for estimating the NB of the proposed model. At a threshold value of z , the *i*th person with a predicted risk of $\pi_{i}$ and correct risk of $p_{\theta i}$ has a probability of $I\left(\pi_{i}>z\right)p_{\theta i}$ for being a true positive and $I\left(\pi_{i}>z\right)(1-p_{\theta i})$ for being a false positive. Thus, if the true value of θ is known, we can consistently estimate the NB of the model as

$$NB_{Model}\left(z;\theta \right)=\frac{1}{n}\sum_{i=1}^{n}\left\{I\left(\pi_{i}>z\right)\left[p_{\theta i}-\left(1-p_{\theta i}\right)\frac{z}{1-z}\right]\right\}.$$

This equation is similar to the equation for $\hat{{NB}_{model}}$ , only that the Y column is replaced with predicted risks from the correct model. As we do not know the true value of θ , in our Bayesian framework, estimating NB at threshold *z* requires taking the expectation with respect to P(θ|D) :

$${\bar{NB}}_{Model}\left(z\right)=E_{\theta }NB_{Model}\left(z;\theta \right).$$

Unlike the conventional estimator for NB, this estimator is the posterior mean in a Bayesian framework, and the frequentist notion of optimism is not directly applicable to it: rather than being based on a single value of θ that might provide an overly good fit to the data, it is the average of NB estimates across the distribution P(θ|D) . Again, using the risk prediction model is not the only option, as we can also either forgo treatment for all or provide treatment to all. The former has zero NB, and the NB for the latter, if the true value of θ is known, can be estimated consistently as

$$NB_{all}\left(z;\theta \right)=\frac{1}{n}\sum_{i=1}^{n}\left[p_{\theta i}-\left(1-p_{\theta i}\right)\frac{z}{1-z}\right],$$

which is, again, the same as $\hat{{NB}_{all}}$ , with the Y column replaced by correct risks. The expected NB of treating all is

$${\bar{NB}}_{all}\left(z\right)=E_{\theta }NB_{all}\left(z;\theta \right).$$

### The EVPI

If we know the correct model, the optimal decision is to use it, instead of the proposed model, for prediction. Indeed, no decision that is based on candidate predictors is more efficient than giving treatment only to those whose correct risk, based on such predictors, is above the threshold. If the true θ is known, the NB of such an optimal strategy can be estimated consistently in the sample as

$$NB_{max}\left(z;\theta \right)=\frac{1}{n}\sum_{i=1}^{n}I\left(p_{\theta i}>z\right)\left[p_{\theta i}-\left(1-p_{\theta i}\right)\frac{z}{1-z}\right].$$

Again, we do not know the true value of θ and instead know about its likely values through P(θ|D) . The expected NB under perfect information is therefore

$${\bar{NB}}_{max}\left(z\right)=E_{\theta }NB_{max}\left(z;\theta \right).$$

On the other hand, without knowing the correct model, the best we can do is to decide whether to use the model, treat no one, or treat all based on their expected NB. The expected NB under current information is therefore $\left\{0,{\bar{NB}}_{model}\left(z\right),{\bar{NB}}_{all}\left(z\right)\right\}$ .

The difference in expected NB with perfect information compared with current information is the expected gain due to knowing the correct model (or expected loss due to not knowing the correct model), which we call the EVPI for model development:

$$EVPI\left(z\right)={\bar{NB}}_{max}\left(z\right)-\max\left\{0,{\bar{NB}}_{Model}\left(z\right),{\bar{NB}}_{all}\left(z\right)\right\}.$$

EVPI is a nonnegative scalar quantity that is in the same unit as the NB for risk models, and its higher values indicate higher expected loss due to prediction uncertainty.

### Relative EVPI

The scale of NB in risk prediction is domain specific, unlike in decision analysis, where NB is typically in the universally interpretable monetary units. As such, the numerical value of EVPI here is the most interpretable in comparison with the expected NB that the model provides. To facilitate this comparison, we suggest a relative version of EVPI. Without using any model, we can choose between treating none or treating all, a decision that confers an expected NB of $\left\{0,{\bar{NB}}_{all}\left(z\right)\right\}$ . This is the “baseline” benefit without any risk stratification. Against this baseline, the expected incremental NB (ΔNB) of using the proposed model is

$${\bar{\Delta NB}}_{current\;information}(z)=max\left\{0,{\bar{NB}}_{Model}\left(z\right),{\bar{NB}}_{all}\left(z\right)\right\}-max\left\{0,{\bar{NB}}_{all}\left(z\right)\right\}.$$

Similarly, the expected ΔNB with knowing the correct risks is

$${\bar{\Delta NB}}_{perfect\;information}\left(z\right)={\bar{NB}}_{max}\left(z\right)-max\left\{0,{\bar{NB}}_{all}\left(z\right)\right\}.$$

The EVPI is the difference between the 2 terms. We suggest the relative EVPI (EVPI_r_) as their ratio:

$$EVPI_{r}\left(z\right)=\frac{{\bar{\Delta NB}}_{perfect\;information}\left(z\right)}{{\bar{\Delta NB}}_{current\;information}\left(z\right)}.$$

This quantity is ≥1 and can be expressed in percentages. An *EVPI_r_* of 1 +*α* means that against the baseline strategy of not using any model, the expected gain in NB with the use of the correct model is α×100% higher than the expected gain in NB with the use of the proposed model. The *EVPI_r_* is +∞ when the denominator is zero but the numerator is positive. This indicates that under current information, the proposed model is not expected to provide extra NB, but the correct model is. Thus, further development might be justified. $\mathrm{EVP}I_{r}$ is undefined when the numerator (and thus the denominator) is zero, but the conclusion is obvious in this case: the correct model and, therefore, the proposed model are not expected to provide extra NB over the default decisions, regardless of current uncertainties.

### A Generic Algorithm for EVPI Calculation Based on Bootstrapping

The Bayesian estimators in the previous sections require taking expectations with respect to P(θ|D) , the posterior distribution of correct model parameters. A fully parametric Bayesian model development approach enables the specification of P(θ|D) given the development data and any prior information. Alternatively, in the conventional likelihood maximization approach in classical regression modeling, P(θ|D) can be derived from the likelihood function. For example, the vector of maximum likelihood estimates of regression coefficients and their estimated covariance matrix from a logistic model specify a multivariate normal distribution as the posterior distribution of regression coefficients under an improper, flat prior. The expectations can then be evaluated using Monte Carlo simulation with repeated sampling from P(θ|D) .

A more flexible approach is to obtain samples from P(θ|D) via bootstrapping. A Bayesian interpretation of the bootstrap enables one to consider a parameter estimate that is derived from a bootstrapped sample as a random draw from the posterior distribution of the parameter given the original sample.^16^ A Bayesian bootstrap of a sample of n i.i.d. observations is obtained by drawing a random vector of weights $\left(w_{1},w_{2},\ldots ,w_{n}\right)$ from a Dirichlet(n;1,1,…,1) distribution.^16^ One way to generate such weights is drawing n−1 standard uniform random variables $u_{1},\ldots ,u_{n-1}$ , ordering them, and calculating the weights as $w_{i}=u_{i}-u_{i-1}$ , where $u_{0}=0$ and $u_{n}=1$ .^16^

The ordinary bootstrap can also be seen as assigning weights to the sample, with weights coming from Multinomial(n;1/n,…,1/n) . The similarity of such weighting approaches has resulted in the ordinary bootstrap being also interpreted in a Bayesian view, as in the imputation of missing data.^17^ Such a bootstrap-based value-of-information approach for decision analysis has been previously proposed, where the Bayesian and ordinary bootstraps and parametric methods generated very similar results.^18^

This bootstrap-based approach for sampling from P(θ|D) provides more flexibility than fully parametric methods, for example, by enabling the incorporation of variable selection and shrinkage and other stochastic steps such as the imputation of missing predictor values. As well, this approach can be embedded with relative ease within the bootstrap-based algorithm for optimism correction. A generic algorithm for EVPI calculation alongside exemplary R code is provided in Table 1. An R package for implementing this methodology (with exemplary code for bootstrap, likelihood-based, and parametric Bayesian approaches for EVPI calculation) is available from https://github.com/resplab/VoIPred.

**Table 1:** Generic Algorithm (Left) and an Exemplary R Implementation (Right) for the Bootstrap-Based EVPI Calculations^a^

1. Using the proposed prediction model, generate the predicted risks for each individual in the development sample ( $\pi_{i}$ s) 2. For *i* = 1 to some large *N* (e.g., 1,000)^b^ and for any threshold *z* in [0,1] 2.1 Obtain a (Bayesian) bootstrap sample from the development data set and perform model development (potentially including variable selection and shrinkage). 2.2 Apply the new model to calculate the predicted risks in the development sample ( $p_{\theta i}$ s). 2.3 Estimate $NB_{Model}\left(z\right)$ , $NB_{all}\left(z\right)$ , and $NB_{max}\left(z\right)$ for this iteration using the predicted risks from the original ( $\pi_{i}$ s) and the new ( $p_{\theta i}$ s) model in the development sample (see the relevant equations in the text). 3. Let ${\bar{NB}}_{Model}\left(z\right)=\mathrm{average}(NB_{Model}(z))$ ; let ${\bar{NB}}_{all}\left(z\right)=\mathrm{average}\left(NB_{all}\left(z\right)\right)$ ; let ${\bar{NB}}_{max}\left(z\right)=\mathrm{average}\left(NB_{max}\left(z\right)\right)$ , with the average taken across all the iterations of the For loop. 4. Calculate (absolute and relative) EVPI(z) .	#Step 0: We develop a simple risk prediction model as an example library(MASS) data(birthwt) n <- dim(birthwt)[1] z <- 0.2 #This is the risk threshold model <- glm(low ~ age + lwt, family=binomial(link="logit"), data= birthwt) #Step 1: pi <- predict(model, type="response") #Predicted risks #Step 2: NBmodel <- NBall <- NBmax <- rep(0,1000) for(i in 1:1000) { #Step 2.1 bsdata <- birthwt[sample(1:n, n, replace = T),] bsmodel <- glm(low ~ age + lwt, family=binomial(link="logit"), data=bsdata) #Step 2.2: #p is a random draw from the distribution of correct risks p <- predict(bsmodel, newdata = birthwt, type="response") #Step 2.3 NBall[i] <- mean(p-(1-p)*z/(1-z)) #NB of treating all NBmodel[i] <- mean((pi>-z)*(p-(1-p)*z/(1-z))) #NB of using the model NBmax[i] <- mean((p>-z)*(p-(1-p)*z/(1-z))) #NB of using the correct risks } #Step 3 ENBall <- mean(NBall); ENBmodel <- mean(NBmodel); ENBmax <- mean(NBmax) #Step 4 EVPI <- ENBmax-max(0,ENBmodel,ENBall) EVPIr <- (ENBmax-max(0,ENBall))/(ENBmodel-max(0,ENBall))

a Expected value of perfect information (EVPI) calculations using methods alternative to the bootstrap are provided in https://github.com/resplab/VoIPred.

b In general, the number of iterations should be high enough such that the Monte Carlo standard error around EVPI is small compared with its point estimate.

### Case Study: Prediction of Mortality after AMI

Identifying the risk of immediate mortality after an AMI can enable stratification of more aggressive treatments for high-risk individuals. GUSTO-I was a large clinical trial of multiple thrombolytic strategies for AMI.^19^ We used data from this study to create a risk prediction model for 30-d mortality after AMI (the primary endpoint of the trial). GUSTO-I’s sample size of 40,830 is larger than typical sizes of development samples in most practical contexts, resulting in a low level of prediction uncertainty.^20^ This provides an opportunity for simulating development samples of smaller sizes that are more typical and studying how EVPI changes as the sample size varies. To start, we assume that we have access to data for only 1000 patients. We randomly selected, without replacement, 1000 individuals from the full sample of GUSTO-I to create such an exemplary development data set. Thirty-day mortality risk was 7.0% in the full sample and 6.7% in this subsample.

In line with previous studies using this data set,^21,22^ our candidate predictors included Killip score (an indicator of heart failure), age, blood pressure, pulse, infarction location, preexisting hypertension, and diabetes. To mitigate the risk of overfitting, we fitted a logistic model via the least absolute shrinkage and selection operator (LASSO), with 10-fold cross-validation to find the optimum shrinkage. Table 2 provides the coefficients of the proposed model. Three candidate predictors were shrunk to zero (not selected) in the final model. To demonstrate uncertainty in regression coefficients, we also report the bootstrap-based 95% confidence intervals and the proportion of bootstraps in which each predictor was selected by LASSO. Confidence intervals, optimism corrections, and EVPI calculations were based on 1000 bootstraps. Computations were performed in R development environment (with *glmnet* package for LASSO).^23^

**Table 2:** Regression Coefficients for the Proposed Model

Predictor	Coefficient^a^	Probability of Selection	95% Confidence Interval
**(Intercept)**	−1.273	1.00	−6.833, 2.744
**Age (y)**	0.050	1.00	0.021, 0.076
**AMI location (other)**	0.259	0.55	−0.034, 1.488
**AMI location (anterior)**	.	0.40	−0.220, 0.521
**History of previous AMI**	0.184	0.63	−0.058, 0.842
**Systolic blood pressure** ^**b**^	−0.070	0.94	−0.104, 0.000
**Killip score >1 (yes v. no)**	0.704	0.97	0.000, 1.277
**Pulse (low)** ^**c**^	0.026	0.75	0.000, 0.038
**Pulse (high)** ^**c**^	.	0.40	0.000, 0.034
**History of hypertension**	.	0.31	−0.587, 0.259
**History of diabetes**	.	0.38	−0.218, 0.664

AMI, acute myocardial infarction.

a Those denoted by ‘.’ are not selected by LASSO.

b This variable was modeled as min(X,100).

c Pulse was modeled using a linear spline with a knot location at 50.

**Figure 1:**
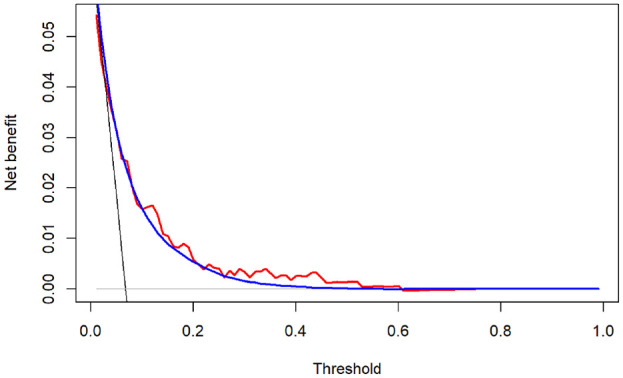
Optimism-corrected (red) net benefit (NB) of the proposed model and its Bayesian estimator (blue), compared with the NB of treating all (black) and treating none (gray). The Bayesian estimation is based on the Bayesian bootstrap (see the relevant section in the text). The optimism correction and Bayesian estimates are based on 1000 bootstraps.

The optimism-corrected c-statistic of the proposed model was 0.758. Figure 1 is the “decision curve” that depicts the optimism-corrected empirical NB ( ${\hat{NB}}_{Model}$ ) of the model (red) alongside those of treating none (gray) and treating all (black). The Bayesian estimator for NB ( ${\bar{NB}}_{Model}$ , blue curve) is also provided. Ordinary and Bayesian bootstraps generated nearly identical results.

**Figure 2:**
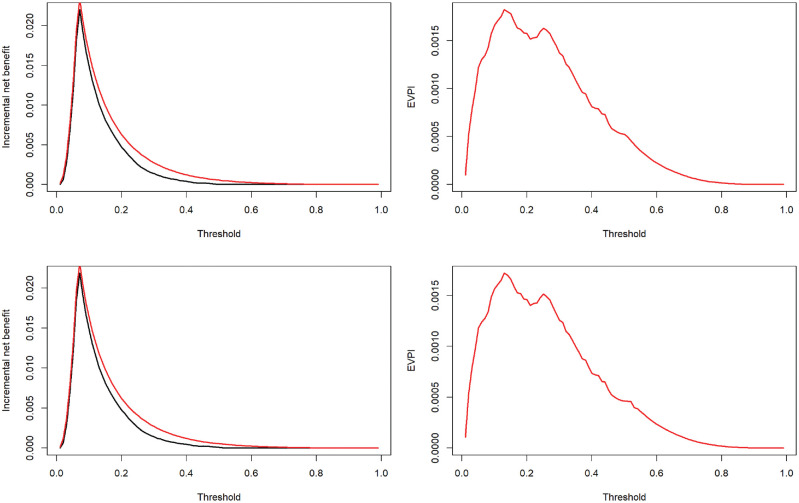
The incremental net benefit curves under current (black) and perfect (red) information (left) and expected value of perfect information (EVPI; right).

Figure 2 depicts the expected incremental NB under current and perfect information (left panels) and EVPI (right panels) at the entire range of thresholds. Results are generated using both ordinary and Bayesian bootstraps, which were very similar. Interpreting the results based on the ordinary bootstrap, at the exemplary threshold of 0.02, the expected NB of treating all was 0.0478, while the expected NB of the model was 0.0484. Thus, the best decision under current information is to use the proposed model, with an expected ΔNB of 0.0006 (black curve in the top-left panel). The expected NB under perfect information was 0.0489, corresponding to an expected ΔNB of 0.0011 (red curve in the top-left panel). Thus, the EVPI is 0.0489 − 0.0484 = 0.0005. The relative EVPI at this threshold is 0.0011/0.0006 = 1.87. That is, knowing the correct prediction model is expected to confer 87% more NB compared with the proposed model. The EVPI is nonzero unless the threshold is unrealistically high (>0.85). The largest gain is obtained within the 0.1–0.3 range. The Bayesian bootstrap generated similar results (EVPI at 0.02 threshold: 0.0005, relative EVPI at this threshold: 1.82).

**Figure 3:**
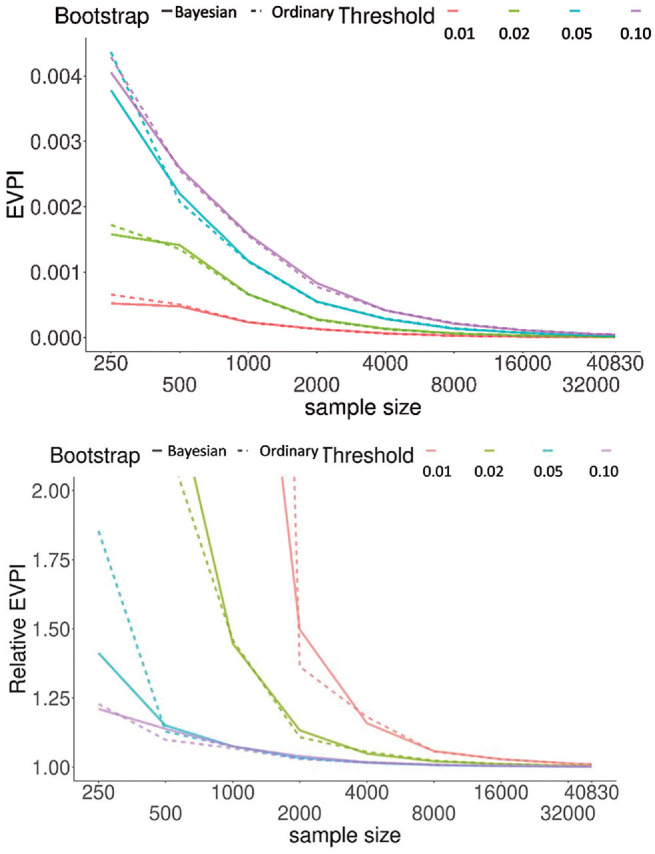
Change in expected value of perfect information (EVPI) (top) and relative EVPI (bottom) as a function of sample size. Results were generated based on randomly obtaining samples, without replacement, of a given size. Results are the average (top) and median (bottom) of 10 independent simulations for each sample size. We discarded data sets with fewer than 8 events as the glmnet optimizer does not reliably converge with too few events. For relative EVPI, the regular bootstrap at 0.01 threshold had a value of 9.7 at sample size 1000; all other truncated lines (reaching >2.0) indicate that the median value was +∞ at smaller sample sizes.

Figure 3 demonstrates how EVPI changes with sample size in GUSTO-I at the exemplary thresholds of 0.01, 0.02, 0.05, and 0.10. We started with *n* = 250 observations and doubled it at each step. For each step, the EVPI (top) and relative EVPI (bottom) were, respectively, the average and median of 10 independent simulations. Both metrics indicated a diminishing gain with larger samples. The median relative EVPI was +∞ for threshold values of 0.01 and 0.02 at *n* = 250 and also at *n* = 500 for the 0.01 threshold. On the other hand, for the model based on the entire GUSTO-I data, the impact of uncertainty was minimal, with EVPI < 0.00001 and relative EVPI = 1.004 at 0.02 threshold. Results of proof-of-concept simulation studies on how EVPI changes with other sample or model characteristics (event probability, model calibration, and discrimination) are provided in the Supplementary Material.

## Discussion

Creating a risk prediction model based on a finite development sample means the resulting predictions are inevitably uncertain. The management plan of a patient based on such predictions might be different from the decision that would have been made had the correct risks been known. As such, prediction uncertainty can result in the loss of NB. We extended the value-of-information methodology from the decision analysis to the development phase of the risk prediction models and applied the definition of EVPI to this context. The proposed development EVPI is a scalar metric that quantifies, for a given risk threshold, the expected loss due to uncertain predictions, with the loss being defined on the same NB scale as is commonly used to assess the utility of the risk prediction models.^11^ In a case study using data from a clinical trial, we demonstrated how EVPI can be calculated and interpreted, for example by determining the range of thresholds within which obtaining a larger development sample could potentially be warranted. We also showed how EVPI behaves when the development sample size is increased. We proposed relative EVPI as a scale-free metric and outlined a generic bootstrap-based algorithm for EVPI calculations that can be embedded within established algorithms for quantifying the optimism of risk prediction models.

How should these developments be used in practice? Once the risk model is developed, the investigators need to decide whether the model is good enough to go to the next stage (i.e., validation), the model should be abandoned, or further model development is required.^9^ Classical arguments in decision theory stipulate that under the conditions of risk neutrality and the absence of irrecoverable costs associated with implementing a health technology, the “adoption decision” and “research decision” are independent: it is solely the expected NB that should determine whether to adopt the model or not,^24^ while value-of-information metrics determine whether further evidence (e.g., obtaining a larger development sample) is required. However, model developers as scientists generally have a preference against seeing their discoveries proven incorrect or harmful,^25^ and patients, care providers, and the general public are on average risk averse.^26,27^ As well, there are significant irreversible costs associated with implementing a risk stratification algorithm only to abandon it later (updating guidelines, incorporating the model into electronic health records). Consequently, uncertainty and the resulting potential for harm become relevant when deciding whether a model should advance to the next stage.

If risk behaviors in the given clinical domain are to be considered, one can update the decision criterion and value-of-information equations with explicit formulation of risk attitudes.^28^ However, in the early phases of model development, investigators might be unwilling to make such judgment calls. We think in this phase what is the most helpful is general guidance on whether the expected loss due to prediction uncertainty is low enough that justifies moving toward model validation. In this context, a zero EVPI indicates that the currently identified best decision is the correct one in this patient population. Similarly, a low EVPI indicates that the potential for harm with current information is small. Such results can motivate model developers to focus on the next stage (e.g., depending on the NB of the model, abandon the model, or move to validation). On the other hand, when the EVPI is large, one should not proceed before an updated model based on a larger development sample is produced. This invokes the question of what value of EVPI is large enough to warrant further model development. Although this is context specific, during the development phase it might make sense to specify thresholds on EVPI as general guidance. For example, an expected loss that is similar to the expected gain by using the proposed model (i.e., relative EVPI ∼2) can be interpreted as the presence of substantial uncertainty and potential for harm. Such a threshold on EVPI can be more relatable than thresholds on statistical metrics such as calibration or shrinkage, whose implications for medical decisions are less clear. This approach can thus potentially lead to stronger consensus among stakeholders and defendable recommendations by authorities who formulate best practice standards in predictive analytics.

The EVPI as defined in this work represents the uncertainty due to the finite development sample, resulting in uncertainty in the regression coefficients of the prediction model. Importantly, this EVPI does not represent the value of knowing the true risk for each individual, which is also a function of predictors that are unknown, unmeasured, or intentionally left out of the model. It also does not include uncertainty due to the potentially systematic differences between the development and the target population (related to external validation which is discussed below). However, modifications of this definition are conceivable that can bring other sources of uncertainty into consideration. Consider, for example, that there is a strong predictor in the development sample that is intentionally excluded because of difficulty in measuring it in practice. If in the Monte Carlo bootstrap algorithm for producing draws from P(θ|D) one includes this predictor in regression models, the resulting EVPI combines the expected loss due to the finite development sample and due to not including the predictor. Similarly, if there are predictors with missing values, incorporating the process of imputing such missing values within Monte Carlo iterations means that the resulting EVPI represents the loss due to the finite development sample and due to missing data.

The Bayesian inference underlying EVPI calculations is based on the assumption that the prior distribution P(θ) and the data model P(D|θ) are compatible with the true data-generating mechanism. Under these assumptions, Bayesian posterior distributions are guaranteed to be calibrated (in contrast with the frequentist inference where a correct model structure by no means prevents overfitting).^29^ These assumptions are similar to the assumptions that enable value-of-information calculations in decision analysis: that the model structure is correct and the probability distributions correctly specify our uncertainty about the values of input parameters. It is indeed improbable that these assumptions are fully met in practice, as both decision-analytic and risk prediction models are simplifications of reality. Nonetheless, value of information in decision analysis is justified based on the working assumption that a model that is good enough for calculating NB is also good enough for quantifying uncertainty around it. We think this assumption is generally a reasonable one in risk modeling. Nevertheless, this framework should be used with caution with black-box algorithms such as machine learning models. Given that such models typically have many free parameters, the cost of model misspecification can be high. In general, to what extent value-of-information quantities are robust against departures from correct model specification needs to be studied.

The application of value of information in risk prediction can be a fruitful endeavor on many fronts. An important area of inquiry is the application of this concept to external validation of risk prediction models. Unlike during model development when the ultimate goal is to identify the correct model, in external validation, the goal is to evaluate whether a prespecified model performs well and thus using it will be beneficial. The expected gain by perfectly knowing if a prespecified model is net beneficial in a new population is different from the expected gain by knowing the correct model in this population. As such, the validation EVPI is distinct from the development EVPI proposed in this article and needs to be pursued independently. Further, the expected value of sample information is a related metric in decision analysis that quantifies the expected gain in NB from conducting a specific study with a given design and sample size.^8^ Defining the equivalent of this metric for risk prediction seems feasible and an immediate extension of the proposed framework. NB calculations have been extended from risk prediction models to models that aim at predicting the benefit of specific interventions,^30^ and value-of-information methods can conceivably be extended to such context.

Contemporary approaches toward evaluating uncertainty in risk prediction target prediction error, calibration, or stability. Despite significant contributions, these metrics are statistical in nature, as they do not relate prediction uncertainty to the outcome of medical decisions. Evaluating the NB of a risk prediction has complemented purely statistical approaches for the assessment of risk prediction models, in a way that is considered a breakthrough in predictive analytics.^9^ We think the assessment of uncertainty in such models can also be augmented with a decision-theoretic perspective.

## Supplemental Material

sj-docx-1-mdm-10.1177_0272989X221078789 – Supplemental material for Uncertainty and the Value of Information in Risk Prediction Modeling Supplemental material, sj-docx-1-mdm-10.1177_0272989X221078789 for Uncertainty and the Value of Information in Risk Prediction Modeling by Mohsen Sadatsafavi, Tae Yoon Lee and Paul Gustafson in Medical Decision Making

## References

[1] van Smeden M Moons KG de Groot JA , et al. Sample size for binary logistic prediction models: beyond events per variable criteria. Stat Methods Med Res. 2019;28(8):2455–74. 10.1177/0962280218784726 PMC6710621 29966490

[2] Riley RD Snell KI Ensor J , et al. Minimum sample size for developing a multivariable prediction model: part II—binary and time-to-event outcomes. Stat Med. 2019;38(7):1276–96. 10.1002/sim.7992 PMC6519266 30357870

[3] Christodoulou E van Smeden M Edlinger M , et al. Adaptive sample size determination for the development of clinical prediction models. Diagn Progn Res. 2021;5(1):6. 33745449 10.1186/s41512-021-00096-5 PMC7983402

[4] Pate A Emsley R Sperrin M Martin GP van Staa T . Impact of sample size on the stability of risk scores from clinical prediction models: a case study in cardiovascular disease. Diagn Progn Res. 2020;4:14. 32944655 10.1186/s41512-020-00082-3 PMC7487849

[5] Buxton MJ Drummond MF Van Hout BA , et al. Modelling in economic evaluation: an unavoidable fact of life. Health Econ. 1997;6(3):217–27. 10.1002/(sici)1099-1050(199705)6:3<217::aid-hec267>3.0.co;2-w 9226140

[6] Felli J Hazen G . Sensitivity analysis and the expected value of perfect information. Med Decis Making. 1998;18(1): 95–109. 9456214 10.1177/0272989X9801800117

[7] Fenwick E Steuten L Knies S , et al. Value of information analysis for research decisions—an introduction: report 1 of the ISPOR Value of Information Analysis Emerging Good Practices Task Force. Value Health. 2020;23(2):139–50. 10.1016/j.jval.2020.01.001 32113617

[8] Ades A Lu G Claxton K . Expected value of sample information calculations in medical decision modeling. Med Decis Making. 2004;24(2):207–27. 10.1177/0272989X04263162 15090106

[9] Kerr KF Marsh TL Janes H . The importance of uncertainty and opt-in v. opt-out: best practices for decision curve analysis. Med Decis Making. 2019;39(5):491–2. 10.1177/0272989X19849436 PMC6786944 31104561

[10] Vickers AJ Cronin AM Elkin EB Gonen M . Extensions to decision curve analysis, a novel method for evaluating diagnostic tests, prediction models and molecular markers. BMC Med Inform Decis Making. 2008;8:53. 10.1186/1472-6947-8-53 PMC2611975 19036144

[11] Vickers AJ Elkin EB . Decision curve analysis: a novel method for evaluating prediction models. Med Decis Making. 2006;26(6):565–74. 10.1177/0272989X06295361 PMC2577036 17099194

[12] Steyerberg EW Harrell FE Borsboom GJ Eijkemans MJ Vergouwe Y Habbema JD . Internal validation of predictive models: efficiency of some procedures for logistic regression analysis. J Clin Epidemiol. 2001;54(8):774–81. 10.1016/s0895-4356(01)00341-9 11470385

[13] Harrell FE . Regression Modeling Strategies: With Applications to Linear Models, Logistic and Ordinal Regression, and Survival Analysis. 2nd ed. Cham (UK): Springer; 2015.

[14] Steyerberg EW Eijkemans MJ Harrell FE Habbema JD . Prognostic modeling with logistic regression analysis: in search of a sensible strategy in small data sets. Med Decis Making. 2001;21(1):45–56. 11206946 10.1177/0272989X0102100106

[15] Van Calster B Nieboer D Vergouwe Y De Cock B Pencina MJ Steyerberg EW . A calibration hierarchy for risk models was defined: from utopia to empirical data. J Clin Epidemiol. 2016;74:167–176. 26772608 10.1016/j.jclinepi.2015.12.005

[16] Rubin DB . The Bayesian bootstrap. Ann Stat. 1981;9(1).

[17] Schafer J . Multiple imputation: a primer. Stat Methods Med Res. 1999;8(1):3–15. 10347857 10.1177/096228029900800102

[18] Sadatsafavi M Marra C Bryan S . Two-level resampling as a novel method for the calculation of the expected value of sample information in economic trials. Health Econ. 2013;22(7):877–82. 10.1002/hec.2869 22933363

[19] GUSTO investigators. An international randomized trial comparing four thrombolytic strategies for acute myocardial infarction. N Engl J Med. 1993;329(10):673–82. 10.1056/NEJM199309023291001 8204123

[20] Steyerberg EW Eijkemans MJC Boersma E Habbema JDF . Applicability of clinical prediction models in acute myocardial infarction: a comparison of traditional and empirical Bayes adjustment methods. Am Heart J. 2005;150(5):920. 10.1016/j.ahj.2005.07.008 16290963

[21] Lee KL Woodlief LH Topol EJ , et al. Predictors of 30-day mortality in the era of reperfusion for acute myocardial infarction: results from an international trial of 41,021 patients. GUSTO-I Investigators. Circulation. 1995;91:1659–68. 10.1161/01.cir.91.6.1659 7882472

[22] Steyerberg EW Borsboom GJJM van Houwelingen HC Eijkemans MJC Habbema JDF . Validation and updating of predictive logistic regression models: a study on sample size and shrinkage. Stat Med. 2004;23(16):2567–86. 10.1002/sim.1844 15287085

[23] R Core Team. R: A Language and Environment for Statistical Computing. Vienna (Austria): R Foundation for Statistical Computing; 2019. Available from: https://www.R-project.org/

[24] Claxton K . The irrelevance of inference: a decision-making approach to the stochastic evaluation of health care technologies. J Health Econ. 1999;18(3):341–64. 10.1016/s0167-6296(98)00039-3 10537899

[25] Parascandola M . Epistemic risk: empirical science and the fear of being wrong. Law Probab Risk. 2010;9(3–4):201–14.

[26] Rosen AB Tsai JS Downs SM . Variations in risk attitude across race, gender, and education. Med Decis Making. 2003;23(6):511–7. 10.1177/0272989X03258431 14672111

[27] Galizzi MM Miraldo M Stavropoulou C van der Pol M . Doctor-patient differences in risk and time preferences: a field experiment. J Health Econ. 2016;50:171–82. 10.1016/j.jhealeco.2016.10.001 27792903

[28] Basu A Meltzer D . Decision criterion and value of information analysis: optimal aspirin dosage for secondary prevention of cardiovascular events. Med Decis Making. 2018;38(4):427–38. 10.1177/0272989X17746988 PMC11379057 29529923

[29] Cook SR Gelman A Rubin DB . Validation of software for Bayesian models using posterior quantiles. J Comput Graph Stat. 2006;15(3):675–92.

[30] Vickers AJ Kattan MW Sargent DJ . Method for evaluating prediction models that apply the results of randomized trials to individual patients. Trials. 2007;8(1):14. 17550609 10.1186/1745-6215-8-14 PMC1914366

