# Supplementary material for: Uncertainty and the Value of Information in Risk Prediction Modeling
Source: Med Decis Making. 2022 Feb 25;42(5):661–71. doi: 10.1177/0272989X221078789 (PMC9194963; doi:10.1177/0272989X221078789)
Supplement: sj-docx-1-mdm-10.1177_0272989X221078789 – Supplemental material for Uncertainty and the Value of Information in Risk Prediction Modeling [file sj-docx-1-mdm-10.1177_0272989X221078789.docx]

Supplementary Material for

Uncertainty and the Value of Information in Risk Prediction Modeling

# Proof-of-concept simulations on the relation between EVPI and sample / model characteristics

In the main text, we showed that EVPI generally declines with increasing sample size. In this section, we further investigate whether EVPI changes ‘intuitively’ to some other features of the sample or the model. The EVPI is the expected loss in net benefit (NB) due to using a proposed model as opposed to the correct model. The intuition is therefore that by increasing the ‘distance’ between the proposed and correct models, the EVPI should increase. To investigate this, in these proof-of-concept simulations we intentionally perturbed the sample or the proposed model and recalculated the EVPI. This perturbance was done on two classical aspects of the model: calibration and discrimination. Generally, miscalibrated models and models with poorer discriminatory performance are further away from the correct model and should have higher EVPI. We also investigated how EVPI changes in response to the event probability as an indicator of the amount of information in the sample. Generally the closer the event probability to 0.5, the higher the ‘effective’ sample size. Thus, we expect that increasing the event probability (from the baseline 0.07 in GUSTO-I) towards 0.5, while holding the sample size constant, will lead to a decrease in EVPI.

***1. Changing calibration-in-the-large while preserving discrimination***

This was performed by changing the intercept of the model. This is equal to applying a fixed odds ratio (OR) to predicted risks. Because OR transformation is a monotonical function, this approach preserves the c-statistic of the model but changes its calibration. We applied OR values of 1/2, 3/4, 4/3, and 2.

***2. Changing discrimination while preserving calibration***

This was achieved by adding a normally distributed random variable (with zero mean and a given standard deviation) to the logit of predicted risks from the proposed model, separately for each predicted risk. Adding such a noise will result in random changes in the ranking of predicted risks, thus reducing model discrimination. However, adding such noise also changes model calibration. As such, we recalibrated the perturbed predicted risks by fitting a logistic regression (with observed response as the dependent variable and the logit of predicted risks from the modified model as the independent variable). The updated predicted risks were the fitted values from this model. Because such transformation is monotonical, the c-statistic remains as before while miscalibration is corrected. We tested scenarios with standard deviation of the error having values 1/3, 2/3, 1, and 3/2.

***3. Changing event probability***

This was performed by assigning weights to each observation. To change the event probability from the observed $p$ to the desired $q$, we assigned relative weights equal to $\frac{q(1-p)}{p\left( 1-q \right)}$ to cases (those experienced the event) and 1 to controls (those who did not experience the event). The weights were then scaled to sum up to the original sample size. We tested event probabilities of 0.15, 0.3, and 0.5.

Results were produced for sample sizes in {500,1000,2500,5000} with ordinary (approximate Bayesian) bootstrap. Each analysis was performed by averaging 10 independent samples and with 200 bootstraps within each iteration.

**Results**

***Table S2*** demonstrates the results. In the majority of scenarios, EVPI changed in the expected direction. An exception was for sample size of 500 at low thresholds were changing the intercept of the model at times reduced its EVPI. This is a reflection of the fact that penalized maximum likelihood estimator in LASSO tries to minimize the off-sample mean square prediction error, which does not necessarily result in a model with the highest NB, and some model perturbations consequently increased the model’s NB (thus reducing the EVPI).

| *Table S2.* Results of simulation studies | | | | | | | | |
| --- | --- | --- | --- | --- | --- | --- | --- | --- |
| Sample  size | Event  probability* | SD of  noise† | Intercept  odds-ratio | EVPI‡ (0.01) | EVPI (0.02) | EVPI (0.05) | EVPI (0.10) | c-  statistic |
| 500 |  |  |  | 0.0005 | 0.0011 | 0.0021 | 0.0026 | 0.842 |
| 500 | 0.15 |  |  | 0.0002 | 0.0006 | 0.0019 | 0.0027 | 0.844 |
| 500 | 0.3 |  |  | 0.0001 | 0.0003 | 0.0010 | 0.0024 | 0.845 |
| 500 | 0.5 |  |  | 0.0000 | 0.0001 | 0.0008 | 0.0020 | 0.844 |
| 500 |  | 1/3 |  | 0.0005 | 0.0013 | 0.0035 | 0.0046 | 0.823 |
| 500 |  | 2/3 |  | 0.0005 | 0.0015 | 0.0043 | 0.0058 | 0.810 |
| 500 |  | 1 |  | 0.0006 | 0.0019 | 0.0061 | 0.0090 | 0.781 |
| 500 |  | 3/2 |  | 0.0006 | 0.0024 | 0.0089 | 0.0123 | 0.741 |
| 500 |  |  | 1/2 | 0.0006 | 0.0025 | 0.0067 | 0.0066 | 0.842 |
| 500 |  |  | 3/4 | 0.0006 | 0.0015 | 0.0033 | 0.0034 | 0.842 |
| 500 |  |  | 4/3 | 0.0004 | 0.0009 | 0.0020 | 0.0034 | 0.842 |
| 500 |  |  | 2 | 0.0004 | 0.0010 | 0.0035 | 0.0076 | 0.842 |
| 1000 |  |  |  | 0.0002 | 0.0005 | 0.0010 | 0.0015 | 0.826 |
| 1000 | 0.15 |  |  | 0.0001 | 0.0002 | 0.0007 | 0.0012 | 0.827 |
| 1000 | 0.3 |  |  | 0.0000 | 0.0001 | 0.0004 | 0.0009 | 0.827 |
| 1000 | 0.5 |  |  | 0.0000 | 0.0000 | 0.0002 | 0.0005 | 0.827 |
| 1000 |  | 1/3 |  | 0.0003 | 0.0010 | 0.0028 | 0.0038 | 0.801 |
| 1000 |  | 2/3 |  | 0.0003 | 0.0013 | 0.0039 | 0.0052 | 0.787 |
| 1000 |  | 1 |  | 0.0004 | 0.0017 | 0.0065 | 0.0086 | 0.754 |
| 1000 |  | 3/2 |  | 0.0005 | 0.0022 | 0.0095 | 0.0129 | 0.708 |
| 1000 |  |  | 1/2 | 0.0005 | 0.0017 | 0.0055 | 0.0056 | 0.826 |
| 1000 |  |  | 3/4 | 0.0003 | 0.0009 | 0.0020 | 0.0023 | 0.826 |
| 1000 |  |  | 4/3 | 0.0002 | 0.0006 | 0.0015 | 0.0024 | 0.826 |
| 1000 |  |  | 2 | 0.0003 | 0.0010 | 0.0038 | 0.0072 | 0.826 |
| 2500 |  |  |  | 0.0001 | 0.0002 | 0.0005 | 0.0007 | 0.817 |
| 2500 | 0.15 |  |  | 0.0000 | 0.0001 | 0.0003 | 0.0005 | 0.817 |
| 2500 | 0.3 |  |  | 0.0000 | 0.0000 | 0.0001 | 0.0004 | 0.817 |
| 2500 | 0.5 |  |  | 0.0000 | 0.0000 | 0.0001 | 0.0002 | 0.818 |
| 2500 |  | 1/3 |  | 0.0002 | 0.0007 | 0.0020 | 0.0031 | 0.717 |
| 2500 |  | 2/3 |  | 0.0003 | 0.0010 | 0.0030 | 0.0046 | 0.705 |
| 2500 |  | 1 |  | 0.0003 | 0.0015 | 0.0052 | 0.0079 | 0.679 |
| 2500 |  | 3/2 |  | 0.0003 | 0.0019 | 0.0082 | 0.0124 | 0.642 |
| 2500 |  |  | 1/2 | 0.0004 | 0.0021 | 0.0046 | 0.0048 | 0.817 |
| 2500 |  |  | 3/4 | 0.0002 | 0.0006 | 0.0012 | 0.0015 | 0.817 |
| 2500 |  |  | 4/3 | 0.0001 | 0.0003 | 0.0010 | 0.0016 | 0.817 |
| 2500 |  |  | 2 | 0.0002 | 0.0009 | 0.0035 | 0.0062 | 0.817 |
| 5000 |  |  |  | 0.0000 | 0.0001 | 0.0002 | 0.0003 | 0.814 |
| 5000 | 0.15 |  |  | 0.0000 | 0.0000 | 0.0001 | 0.0003 | 0.814 |
| 5000 | 0.3 |  |  | 0.0000 | 0.0000 | 0.0001 | 0.0002 | 0.815 |
| 5000 | 0.5 |  |  | 0.0000 | 0.0000 | 0.0000 | 0.0001 | 0.815 |
| 5000 |  | 1/3 |  | 0.0002 | 0.0008 | 0.0026 | 0.0034 | 0.790 |
| 5000 |  | 2/3 |  | 0.0003 | 0.0011 | 0.0037 | 0.0048 | 0.777 |
| 5000 |  | 1 |  | 0.0004 | 0.0017 | 0.0061 | 0.0078 | 0.750 |
| 5000 |  | 3/2 |  | 0.0004 | 0.0022 | 0.0094 | 0.0127 | 0.710 |
| 5000 |  |  | 1/2 | 0.0004 | 0.0021 | 0.0044 | 0.0047 | 0.814 |
| 5000 |  |  | 3/4 | 0.0002 | 0.0005 | 0.0010 | 0.0012 | 0.814 |
| 5000 |  |  | 4/3 | 0.0001 | 0.0002 | 0.0007 | 0.0013 | 0.814 |
| 5000 |  |  | 2 | 0.0002 | 0.0008 | 0.0031 | 0.0059 | 0.814 |
| ** Empty cells indicate simple random sampling without changing event probability*  *†* *SD of the normally distributed random variable that was added to the logit of predicted risk for each observation*  *‡ Number in parentheses is the risk threshold*  *EVPI: Expected Value of Perfect Information;* SD: standard deviation | | | | | | | | |
